# *In Vivo* Induction of Tr1 Cells via Mucosal Dendritic Cells and AHR Signaling

**DOI:** 10.1371/journal.pone.0023618

**Published:** 2011-08-23

**Authors:** Henry Yim Wu, Francisco J. Quintana, Andre Pires da Cunha, Benjamin T. Dake, Thomas Koeglsperger, Sarah C. Starossom, Howard L. Weiner

**Affiliations:** Center for Neurologic Diseases, Department of Neurology, Brigham and Women's Hospital, Harvard Medical School, Boston, Massachusetts, United States of America; Heart Center Munich, Germany

## Abstract

**Background:**

Type 1 regulatory T (Tr1) cells, characterized by the secretion of high levels of the anti-inflammatory cytokine interleukin-10 (IL-10), play an important role in the regulation of autoimmune diseases and transplantation. However, effective strategies that specifically induce Tr1 cells *in vivo* are limited. Furthermore, the pathways controlling the induction of these cells *in vivo* are not well understood.

**Methodology/Principal Findings:**

Here we report that nasal administration of anti-CD3 antibody induces suppressive Tr1 cells in mice. The *in vivo* induction of Tr1 cells by nasal anti-CD3 is dependent on IL-27 produced by upper airway resident dendritic cells (DCs), and is controlled by the transcription factors aryl hydrocarbon receptor (AHR) and c-Maf. Subsequently, IL-21 acts in an autocrine fashion to expand and maintain the Tr1 cells induced *in vivo* by nasally administered anti-CD3.

**Conclusions/Significance:**

Our findings identify a unique approach to generate Tr1 cells *in vivo* and provide insights into the mechanisms by which these cells are induced.

## Introduction

The generation of functional regulatory T cells in vivo is a major goal for the treatment of immune-mediated diseases. Tr1 cells are regulatory T cells characterized by a cytokine profile that is distinct from T helper 1 (Th1), Th2, Th3 and Foxp3+ regulatory T cells (Treg) [1]. Tr1 cells do not constitutively express the transcription factor forkhead box p3 (Foxp3), which is a lineage specific marker for both naturally occurring and induced CD4+CD25+ regulatory T cells [2]. Upon T-cell receptor (TCR) mediated activation, Tr1 cells produce high levels of IL-10 and transforming growth factor-beta (TGF-β), low levels of interferon-gamma (IFN-γ) and almost no IL-2 or IL-4.

The mechanism of in vitro suppression by Tr1 cells is linked to IL-10 [3], [4] as neutralization of IL-10 by monoclonal antibodies typically reverses suppression. Upon TCR stimulation, Tr1 cells can mediate bystander suppression by the local release of IL-10 and TGF-β that act on both antigen presenting cells (APCs) and T cells to suppress co-stimulatory molecule expression and pro-inflammatory cytokine production, respectively [5].

Tr1 cells can be generated in vitro from naïve precursors in response to different cytokine milieus. Early studies in which antigen-specific Tr1 cells were induced in vitro by repeated TCR stimulation in the presence of high doses of IL-10 suggested that IL-10 plays an important role in Tr1 cell differentiation [1]. However, it has been recently shown that IL-10 does not play a crucial role during the differentiation of Tr1 cells in vivo [6]. We [7] and others [8] have identified a critical function for IL-27 in the induction of Tr1 cells. Specifically, we found that DC-derived IL-27 is required for the differentiation of IL-10-secreting Tr1 cells, this process is amplified by TGF-β [6], [7].

Although the generation of Tr1 cells potentially constitutes a new therapeutic approach for immune-mediated diseases, methods for the induction of Tr1 cells in vivo are still missing. Here we report that nasal anti-CD3 triggers the differentiation of suppressive Tr1 cells by a mechanism dependent on the production of IL-27 by upper airway-resident DCs. Furthermore, the generation of Tr1 cells in vivo is controlled by AHR and c-Maf in T cells, and the autocrine effects of IL-21. Thus, nasally administered anti-CD3 might constitute a new approach for the therapeutic induction of Tr1 cells.

## Results

### Nasal administration of anti-CD3 induces suppressive Tr1 cells

**Figure 1:**
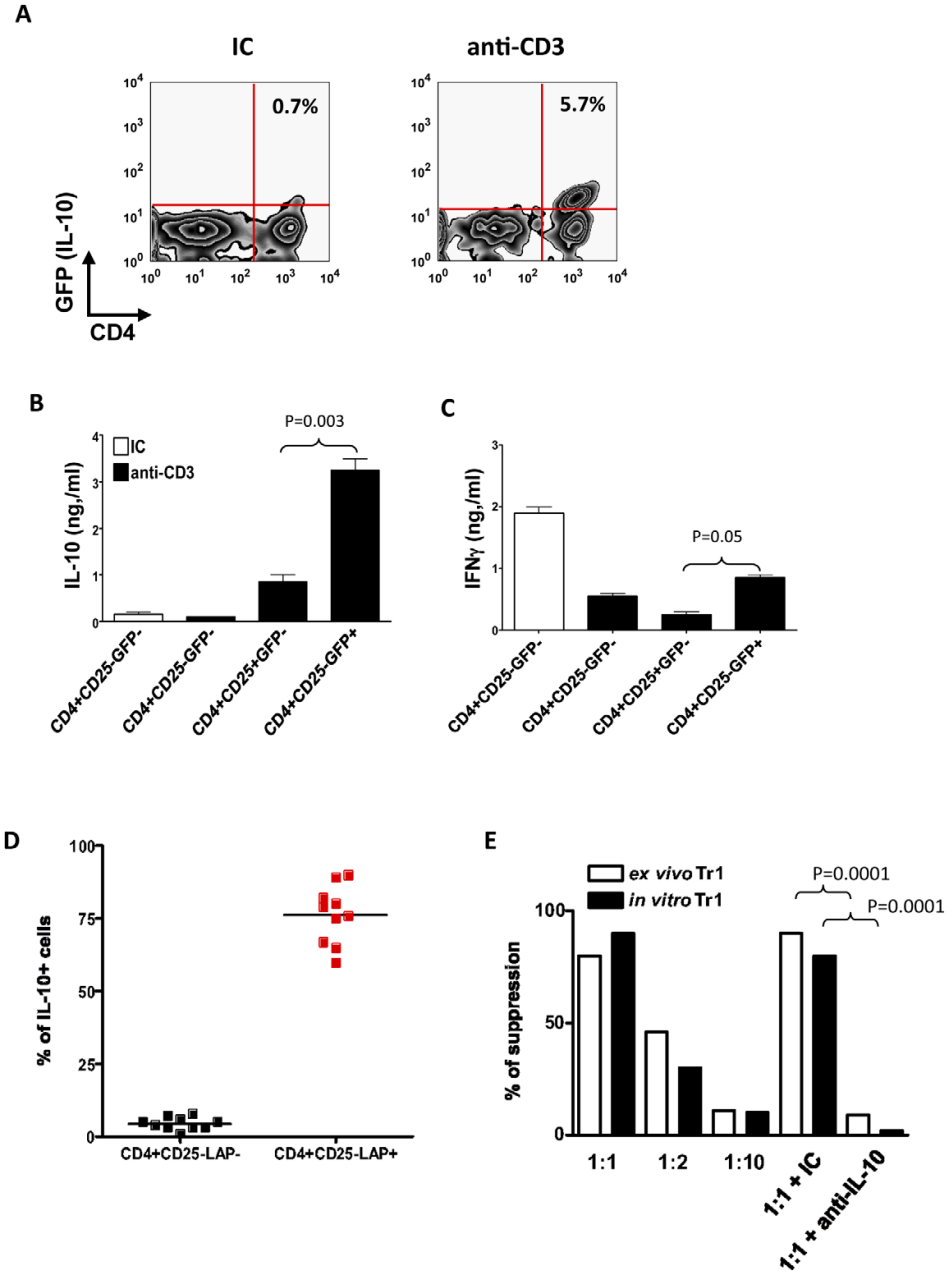
Nasal anti-CD3 induces suppressive Tr1 cells. **A.** Tiger mice were nasally treated with IC (clear bars) or anti-CD3 (filled bars) and 72 hrs after the last nasal dose GFP(IL-10) expression by CD4+ T cells in CLN was examined by flow cytometry. This experiment was repeated 4 times with same results. **B and** **C.** CD4+CD25-GFP-, CD4+CD25+GFP- or CD4+CD25-GFP+ T cells were sorted from CLN of Tiger mice nasally treated with IC (clear bar) or anti-CD3 (filled bar). Sorted T cells were stimulated in vitro with plate bound anti-CD3 and anti-CD28 antibodies (1 µg/ml each) and IL-10 (**B**) and (**C**) IFN-γ were detected in the supernatants by ELISA. Error bars represent standard deviations and P values were calculated by t-test. **D.** The percentage of CD4+CD25-LAP+ T cells that express IL-10 following nasal anti-CD3 was assessed by intracellular staining. Each symbol represents an individual mouse. **E**. FACS-sorted Tr1 cells (CD4+CD25-GFP(IL-10)+, clear bar) from CLN of nasal anti-CD3 treated Tiger mice or IL-27 in vitro differentiated Tr1 cells (filled bar) were used in a standard suppression assay with naïve CD4+CD25-GFP- responder T cells at various ratios. To test the role of IL-10 in in vitro suppression, IC or anti-IL-10 (50 µg/ml) neutralizing antibodies were added to co-cultures at 1∶1 ratio.

We used tiger mice [9] carrying a green fluorescent reporter (GFP) reporter inserted immediately before the polyadenylation site of the *il10* gene to investigate the effect of nasal administration of anti-CD3 on CD4+ IL-10+ T cells. We found that the frequency of CD4+CD25-GFP(IL-10)+ cells was upregulated following nasal treatment with anti-CD3 (**Figure 1A**). Upon activation with anti-CD3 in vitro, FACS sorted CD4+CD25-GFP(IL-10)+ T cells secreted IL-10 and IFN-γ (**Figure 1B**). This cytokine pattern is consistent with a Tr1 cell phenotype [10], and was not seen when CD4+CD25-GFP(IL-10)- naive T cells or CD4+CD25+GFP(IL-10)- T cells were sorted from anti-CD3 treated mice and activated in vitro (**Figure 1B**).

We have previously shown that the suppressive T cells induced by the oral administration of anti-CD3 are characterized by the expression of membrane-bound TGF-β (LAP). In accordance with our previous observations, we found that the CD4+CD25-GFP(IL-10)+ T cells induced by the nasal administration of anti-CD3 were mostly LAP+ (**Figure 1c**).

We next studied the suppressive activity of the CD4+CD25-GFP(IL-10)+ T cells induced by nasal treatment with anti-CD3. We found that CD4+CD25-GFP(IL-10)+ T cells isolated from anti-CD3 treated mice suppressed the proliferation of responder CD4+CD25-GFP- T cells (**Figure 1D**). The suppressive activity of the CD4+CD25-GFP(IL-10)+ T cells induced by the nasal administration of anti-CD3 was mediated by IL-10, because it could be abrogated with IL-10 specific antibodies (**Figure 1D**). Similar results were observed when we analyzed the suppressive activity of CD4+ GFP(IL-10)+ Tr1 cells induced in vitro with IL-27 (**Figure 1D**). Taken together these data demonstrate that nasal anti-CD3 generates suppressive LAP+ Tr1 cells.

### IL-27 secreted by upper airway-resident DCs is required for the induction of Tr1 cells by nasal anti-CD3

**Figure 2:**
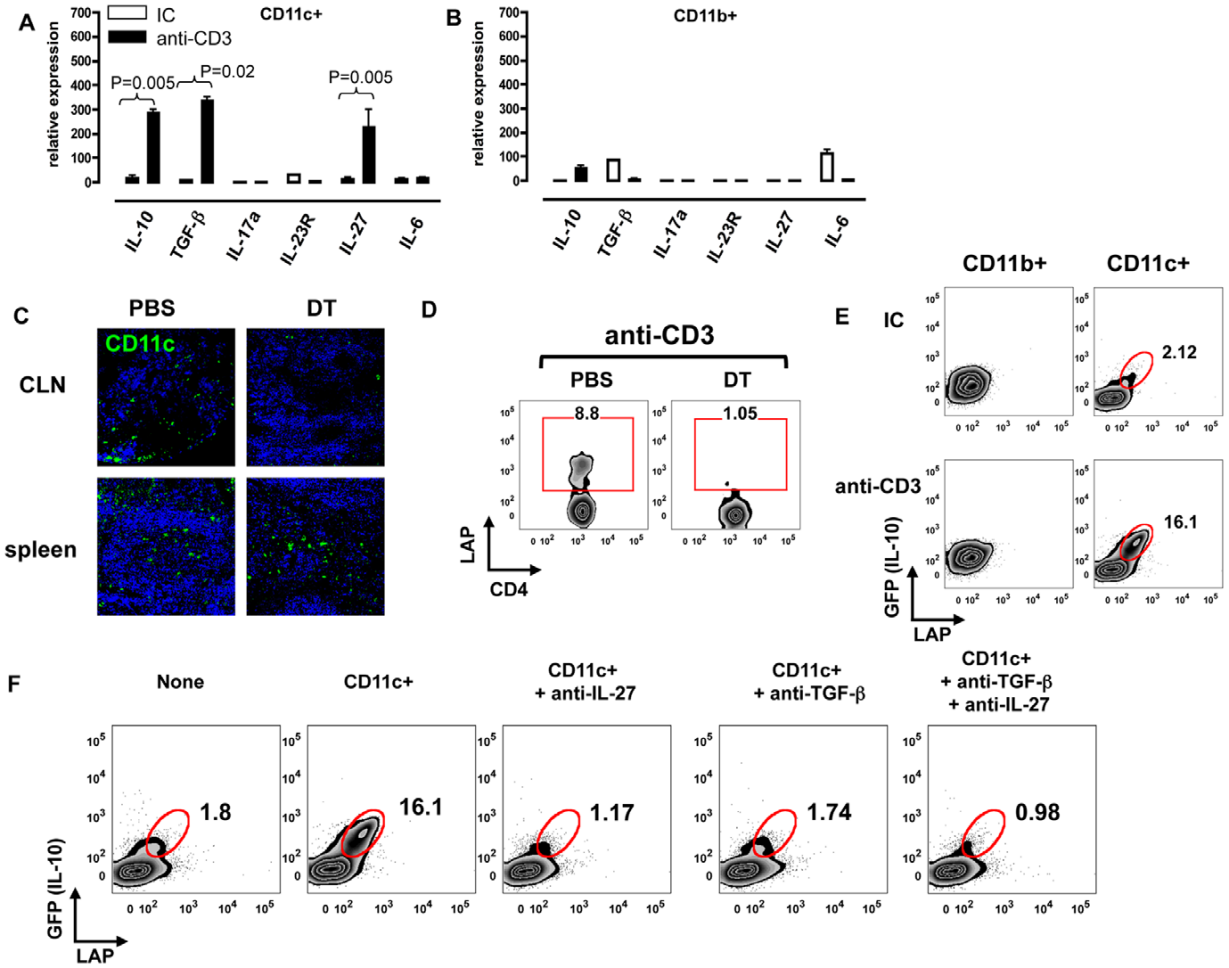
In vivo induction of Tr1 cells is dependent on mucosal DC-derived IL-27 and TGF-β. **A.** CD11c+ DCs or **B.** CD11b+ macrophages were positively selected from CLNs following nasal IC (clear bars) or anti-CD3 (filled bars) and used in quantitative RTPCR. This experiment was repeated 3 times with same results. Error bars represent standard deviations and P values were calculated by t-test. **C.** CD11c DTR-GFP mice were nasally treated with PBS or 500 ng of DT. CLNs and spleens were harvested 24 hrs following nasal DT. 10 µM frozen sections were stained with Toprol-3. Pictures were taken at ×40 magnification. **D.** CD11c DTR-GFP mice were nasally treated with anti-CD3 or DT followed anti-CD3. 72 hrs following nasal treatment, CLN cells were stained with anti-CD4, anti-CD25 and anti-LAP antibodies for FACS. LAP staining on gated CD4+CD25- T cells is shown. **E.** B6 mice were nasally treated with anti-CD3. CD11b+ macrophages (left column) or CD11c+ DCs (right column) were positively selected from CLN at 72 hrs after the last nasal dose. Co-cultures of macrophages or DCs and CD4+CD25-GFP- naïve Tiger T cells were stimulated with LPS (1 µg/ml) or FLT3 ligand (1 µg/ml) respectively and plate bound anti-CD3 (1 µg/ml) for 96 hrs. Cells were stained with anti-LAP antibody. FACs plots shown here were on gated CD4+ lymphocytes. Representative FACs plots of 3 independent experiments are shown here. **F.** B6 mice were nasally treated with anti-CD3. CD11c+ DCs were positively selected from CLN 72 hrs after the last nasal dose. Co-cultures of DCs and CD4+CD25-GFP- naïve Tiger T cells were stimulated with 1 µg of FLT3 ligand and plate bound anti-CD3 in the presence of 10 µg/ml neutralizing antibody to TGF-β and/or IL-27 for 96 hrs. Cells were stained with anti-LAP antibody. Representative FACs plots of 3 independent experiments are shown here.

DCs play an important role in the activation and polarization of T cells in vivo [11]. Indeed, we and others have recently described that DC-derived IL-27 [7], [8] and TGF-β[6], [7] play a critical role for in the differentiation of Tr1 cells. To investigate the role of DCs in the generation of Tr1 cells in vivo, we studied the effect of nasal anti-CD3 on the production of cytokines by CD11c+ and CD11b+ cells in the cervical lymph node (CLN). We found that nasal administration of anti-CD3 induces a unique cytokine profile in CD11c+ DCs, characterized by the expression of IL-10, TGF-β and IL-27 (**Figure 2A**). This profile was not observed in CD11b+ macrophages (**Figure 2B**) or splenic or mesenteric lymph node-derived DCs (not shown).

We thus examined the requirement for upper airway resident DCs in the generation of IL-10-secreting LAP+ Tr1 cells, using transgenic mice in which the CD11c promoter controls the expression of a diphtheria toxin receptor (DTR)-GFP cassette [12]. Nasal administration of diphtheria toxin (DT) led to a significant depletion of CD11c+ DCs in the CLN but not in spleen (**Figure 2C**). Moreover, depletion of CLN resident DCs by nasal DT abolished the generation of the IL-10-secreting CD4+CD25-LAP+ T cells induced by nasal anti-CD3 (**Figure 2D**).

To further investigate the role of DCs in the differentiation of IL-10-secreting Tr1 cells, we co-cultured naïve (CD4+CD25-GFP-) Tiger T cells with CD11c+ DCs or CD11b+ macrophages harvested from the CLN of mice treated with nasal anti-CD3. Co-incubation with DCs from mice treated with nasal anti-CD3 upregulated the expression of GFP (IL-10) and LAP in T cells (**Figure 2E**). We then used neutralizing antibodies to IL-27 and/or TGF-β to investigate the mechanisms of Tr1 induction by DCs taken from anti-CD3 treated mice. We found that the induction of GFP (IL-10) and LAP expression by Tr1 cells in vitro was dependent both on DC-derived IL-27 and TGF-β signaling as neutralizing antibodies to IL-27 and/or TGF-β suppressed the expression of both GFP (IL-10) and LAP by Tr1 cells (**Figure 2F**). Taken together, these findings suggest that local DCs promote the generation of LAP+ Tr1 cells by nasal anti-CD3 via the secretion of IL-27 and TGF-β.

### Tr1 cells induced by nasal anti-CD3 express *ahr*, *cmaf*, *il21* and *il21r*

**Figure 3:**
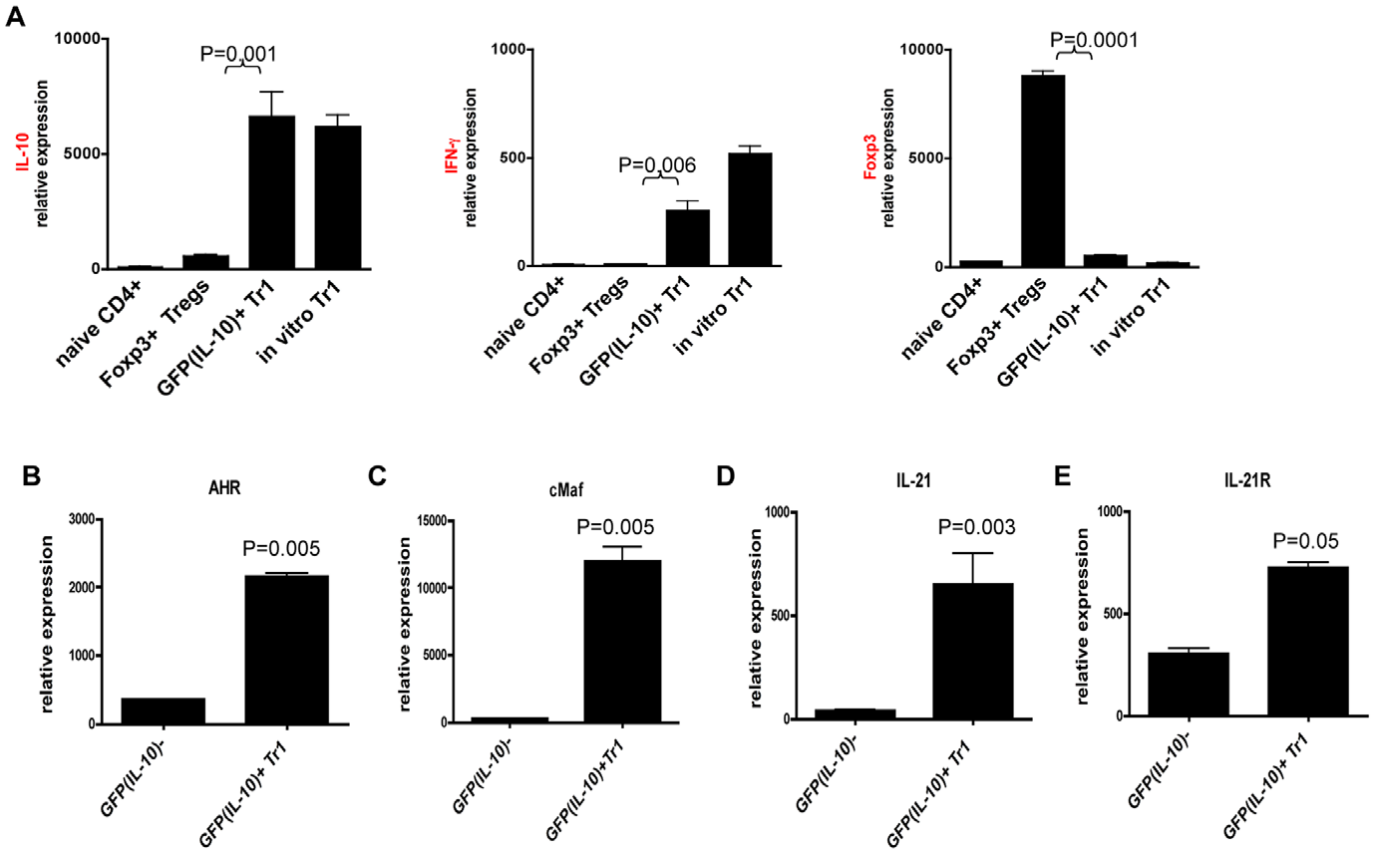
Tr1 cells induced by nasal anti-CD3 express ahr, cmaf, il21 and il21r. **A.** Naive CD4+ T cells (CD4+CD25-GFP(IL-10)-), nTregs (CD4+CD25+GFP(foxp3)+) sorted from Foxp3-GFP knock-in mice, ex vivo Tr1 cells (CD4+CD25-GFP(IL10)+) sorted from CLN of nasal anti-CD3 treated Tiger mice and in vitro differentiated Tr1 using plate bound anti-CD3 and anti-CD28 plus 50 ng/ml IL-27 were used in quantitative RTPCR reactions. Expressions of IL-10, IFN-γ and foxp3 mRNA were normalized to expression of β-actin. **B.** AHR, **C.** cMAF, **D.** IL-21 and **E.** IL-21R mRNA expression by CD4+GFP(IL-10)- T cells or CD4+GFP(IL-10)+ Tr1 cells. These experiments were repeated 3 times with same results.

The transcription factor c-Maf plays an important role in the regulation of *il10* expression [13], [14], [15], [16]. We have recently shown that c-Maf interacts with the transcription factor AHR to control the expression of *il10* and the autocrine Tr1 growth factor *il21* [*17*]. AHR and cMAF also cooperate to control the expression of human *IL10* [18]. To investigate the molecular mechanisms leading to the differentiation of Tr1 cells in vivo in response to the nasal administration of anti-CD3, we studied the expression of *maf*, *ahr*, *il21* and *il21r*; was also analyzed the expression of *il10*, *ifng* and *foxp3*. Naïve T cells and CD4+CD25-GFP(IL-10)+ Tr1 cells were FACS-sorted from tiger mice treated with nasal anti-CD3, Foxp3+ nTregs were isolated from Foxp3 GFP knock-in transgenic mice [19], and gene expression was analyzed by quantitative PCR. We found that freshly isolated CD4+CD25-GFP(IL-10)+ Tr1 cells induced by treatment with nasal anti-CD3 consistently expressed high levels of *il10* and also some *ifng*, however *foxp3* expression was undetectable (**Figure 3A**). Moreover, freshly isolated Tr1 cells from nasal anti-CD3 treated mice expressed significant levels of *ahr* (**Figure 3B**) and *maf* (**Figure 3C**) as well as *il21* (**Figure 3D**) and *il21r* (**Figure 3E**). Thus the Tr1 cells induced by treatment with nasal anti-CD3 express the transcription factors AHR, c-Maf, and also the autocrine growth factor IL-21.

### AHR signaling and IL-21 mediate the induction of Tr1 cells by nasal anti-CD3

**Figure 4:**
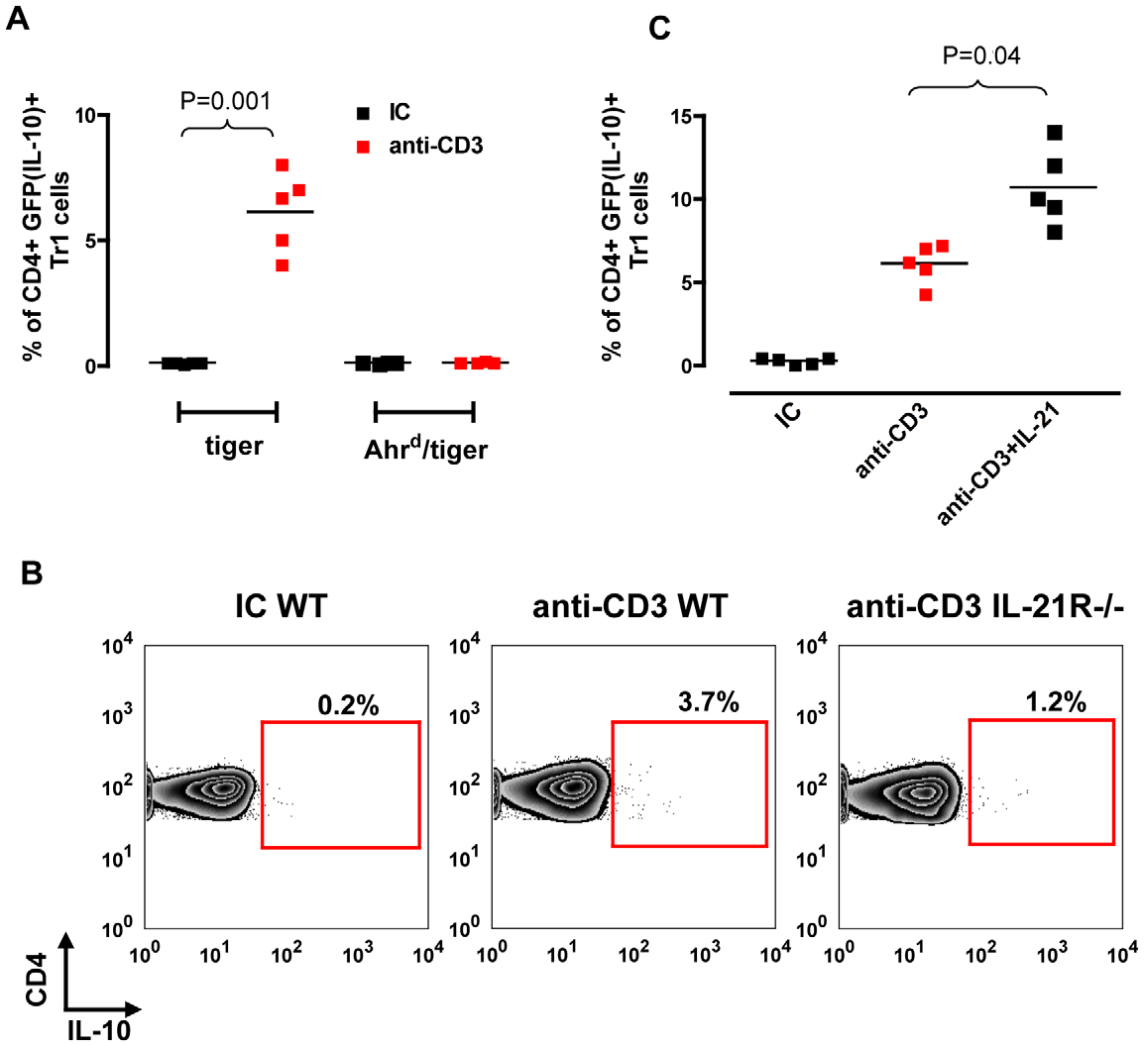
The induction of Tr1 cells by nasal anti-CD3 requires AHR and IL-21 signaling. **A.** Tiger or Ahr^d^/tiger mice were nasally treated with IC or anti-CD3 and 72 hrs after the last nasal dose GFP(IL-10) expression by CD4+ T cells in CLN was examined by flow cytometry. Each symbol represents an individual mouse. **B.** Tiger mice were nasally treated with IC or anti-CD3 alone or together with recombinant mouse IL-21 (4 µg/day). **C.** WT or IL-21R−/− mice were nasally treated with IC or anti-CD3.

To investigate the role of AHR in the generation of Tr1 cells in vivo we generated tiger mice carrying a mutant AHR protein that shows a reduced affinity for its ligands (*Ahr^d^*) [20]. We thus studied the frequency of Tr1 cells in the CLN following nasal anti-CD3 in tiger and *Ahr^d^*/tiger mice. **Figure 4A** shows that induction of GFP(IL-10)+ Tr1 cells by nasal anti-CD3 is completely abolished in the *Ahr^d^*/tiger mice, thus AHR signaling is essential for the generation of Tr1 cells in vivo in response to nasally administered anti-CD3.

IL-21 is an autocrine growth factor for Tr1 cells [21], [22]. We have recently reported that AHR and c-Maf directly control the production of IL-21 during the differentiation of Tr1 cells [17]. Thus, based on the expression of *il21* and *il21r* by Tr1 cells induced in response to the nasal administration of anti-CD3, we investigated the role of IL-21 in the generation of Tr1 cells in vivo. We first studied the effect of treating tiger mice with anti-CD3 co-administered nasally with recombinant IL-21 or vehicle as control. We found that the nasal co-administration of anti-CD3 with recombinant IL-21 led to a significant increase in the generation of Tr1 cells (**Figure 4B**). We then analyzed the induction of Tr1 cells by nasal anti-CD3 in IL-21R −/− and wild type mice. We found a significant impairment in the induction of Tr1 cells triggered by nasal anti-CD3 in IL-21R −/− mice (**Figure 4C**). Thus, IL-21 plays an important role in the induction of Tr1 cells in vivo as a result of treatment with nasal anti-CD3.

### Tr1 cells induced by nasal anti-CD3 in an IL-27-dependent manner control systemic autoimmunity

**Figure 5:**
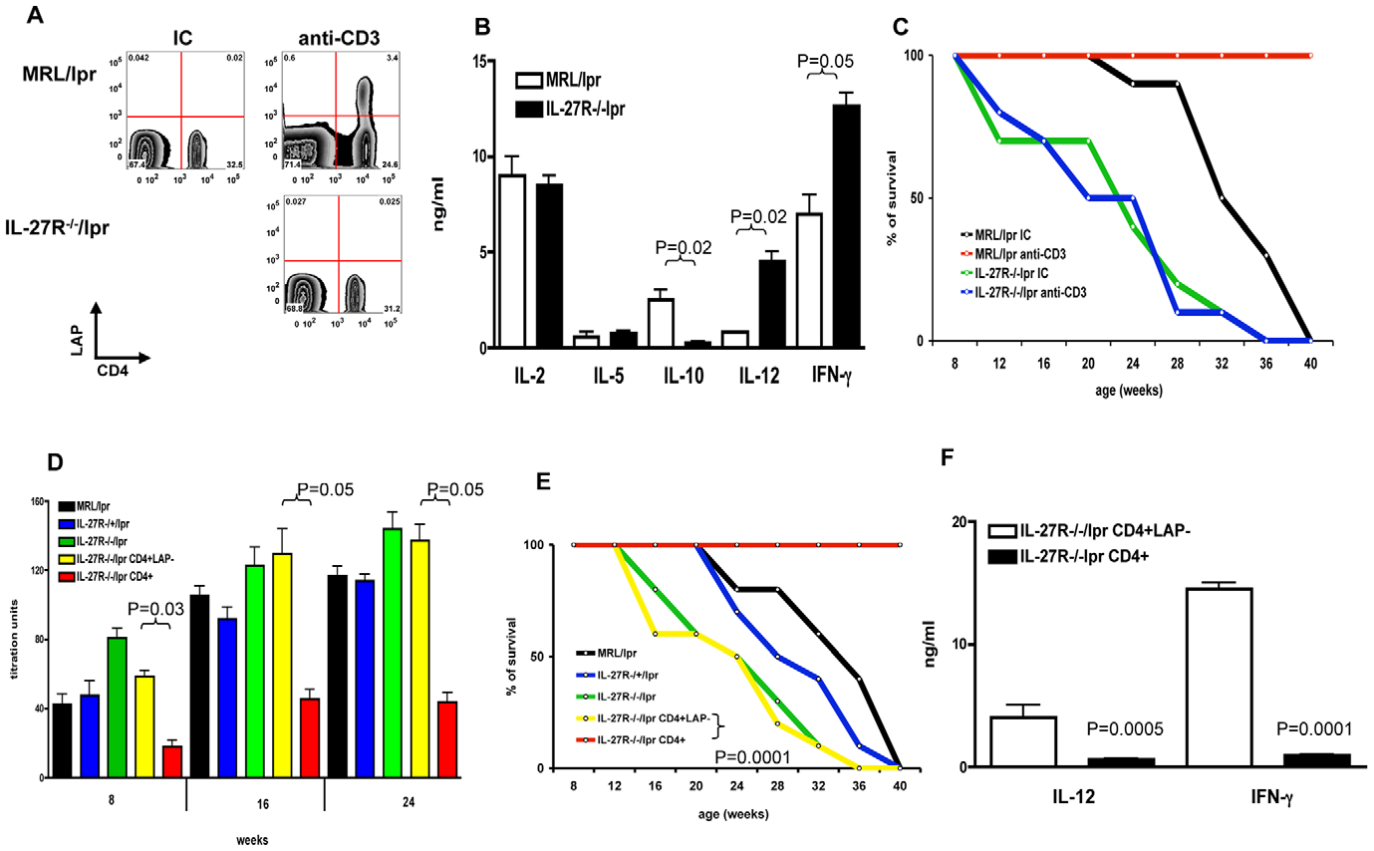
Tr1 cells induced by nasal anti-CD3 in an IL-27-dependent manner control systemic autoimmunity. **A.** 8 wks old female MRL/lpr or IL-27R−/−/lpr mice were nasally treated with 0.5 µg IC or anti-CD3. CLN cells were harvested at 72 hrs after the last nasal dose and stained with anti-CD4 and anti-LAP antibodies. **B.** CD4+ T cells were isolated by positive selection and stimulated with plate bound anti-CD3 and anti-CD28 antibodies (1 µg/ml each) for 96 hrs. Culture supernatant was used in detection of IL-2, IL-5, IL10, IL-12 and IFN-γ by ELISA. **C.** 4-wk old MRL/lpr or IL-27R−/−/lpr mice (n = 10) received three 5-day courses of 0.5 µg IC or anti-CD3 given at alternative weeks. Survival of mice following treatment was followed for 40wks. **D.** Serum was collected from MRL/lpr, IL-27R−/+/lpr, IL-27R−/−/lpr or IL-27R−/−/lpr recipients of CD4+LAP- or CD4+ T cells. IgG anti-dsDNA autoantibodies were detected by ELISA. **E.** 8 wk old female MRL/lpr mice were nasally treated with anti-CD3 for 5 consecutive days. 72 hrs after the last nasal dose CD4+ (red line) or CD4+LAP- (yellow line) T cells were sorted from CLN cells and adoptively transferred (1×10^6^ cells/mouse) to 4-wk old IL-27R−/−/lpr recipients (n = 10). Survival of recipients was followed for 40wks and compared to MRL/lpr (black line), IL-27R−/+/lpr (blue line) or IL-27R−/−/lpr (green line) mice (n = 10) without cell transfer. **F.** CD4+ T cells from IL-27R−/−/lpr recipients of CD4+LAP- (clear bars) or CD4+ (filled bars) T cells were stimulated with plate bound anti-CD3 and anti-CD28 antibodies for 96 hrs. IL-12 and IFN-γ in culture supernatant was detected by ELISA.

To investigate the role of IL-27 in the induction of Tr1 cells by nasal anti-CD3, we backcrossed IL-27 receptorα-deficient mice onto the lupus prone *lpr* background (IL-27R−/−/lpr). **Figures 5A and 5B** show that the generation of LAP+ Tr1 cells following nasal administration of anti-CD3 is dependent on IL-27, as no upregulation of LAP was seen in IL-27R−/−/lpr mice. Concomitant with the deficient generation of LAP+ Tr1 cells, we found that CD4+ T cells from IL-27R−/−/lpr mice given nasal anti-CD3 produced significantly higher amounts of IFN-γ and IL-12 upon in vitro stimulation with anti-CD3 (**Figure 5B**).

We next investigated the role of IL-27 and LAP+ Tr1 cells induced by nasal anti-CD3 in the control of systemic autoimmunity. IL-27R−/−/lpr mice developed spontaneous fatal autoimmunity significantly earlier than MRL/lpr mice (**Figure 5C**, compare green and black lines). This accelerated development of fatal autoimmunity was associated with a progressive increase in serum IgG autoantibodies to double stranded DNA (dsDNA) (**Figure 5D**). Moreover, nasal anti-CD3 (three 5-day courses of 0.5 µg/day given at alternative weeks) significantly prolonged the survival of MRL/lpr but not of IL-27R−/−/lpr mice (**Figure 5C**, compare red and blue lines). Taken together, these data suggests that IL-27 is required for the generation of suppressive LAP+ Tr1 cells following nasal administration of anti-CD3.

To confirm that the lack of protective effect of nasal anti-CD3 in IL-27R−/−/lpr mice is linked to the defective induction of LAP+ Tr1 cells (**Figures 5A and 5B**) we performed adoptive transfer experiments. The transfer of CLN CD4+ T cells from wild type mice treated with nasal anti-CD3 protected IL-27R−/−/lpr recipients from the development of fatal autoimmunity (**Figure 5E**, compare blue and red lines). This protection was associated with a significant reduction in the production of IgG anti-dsDNA autoantibodies (**Figure 5D**). The suppression of systemic autoimmunity in this adoptive transfer system was dependent on LAP+ Tr1 cells, as protection was reversed by depletion of LAP+ T cells prior to cell transfer (**Figure 5E**, compare yellow and red lines).

We found similar effects when the recipients of LAP+ Tr1 cells were investigated in terms of autoantibody production (**Figure 5D**) and IFN-γ and IL-12 production (**Figure 5F**). Thus, IL-27 mediates the induction of LAP+ suppressive Tr1 cells following the nasal administration of anti-CD3 in the context of ongoing inflammation.

## Discussion

Tr1 cells are regulatory T cells that do not express Foxp3 and suppress tissue inflammation, graft-versus-host disease and autoimmunity in an IL-10 dependent manner. IL-27 plays a major role in the differentiation of IL-10-secreting Tr1 cells [7], [23]. Indeed, we have recently demonstrated that IL-27 induces the expression of the transcription factors AHR and c-Maf, which cooperate to control the expression of *il10* and of the autocrine Tr1 growth factor *il21* [17]. AHR and cMaf also cooperate to control the expression of human *Il10* [*18*].

Here we show that nasal administration of an anti-CD3 monoclonal antibody induces suppressive Tr1 cells. We found that the induction of Tr1 cells is dependent on local DCs that express IL-27, IL-10 and TGF-β as site-specific depletion of DCs abolishes Tr1 cell generation by nasal anti-CD3. To our knowledge, this is the first demonstration of an essential role of upper airway-resident DCs in the generation of Tr1 cells in vivo. Accumulating evidence suggests that resident DCs in mucosal tissues possess unique features not shared by DCs in peripheral lymphoid tissues [24]. Most notably is their ability to generate regulatory T cells that suppress airway and gut inflammation in mouse models of asthma [25] and inflammatory bowel disease [26]. In support of these findings, it has been demonstrated that resident DCs in lamina propria and mesenteric lymph nodes are critical for the generation of Foxp3+ regulatory T cells in the gut [27], [28], [29].

The upper airway mucosal DCs may be controlled by local signals produced by the bronchial and intestinal epithelia. For instance, in co-culture studies it has been shown that products of epithelial cells condition DCs to promote Th2 immunity in an allogeneic response [30]. It is possible that in the face of continuous challenge from environmental antigens, mucosal epithelial cells secrete cytokines that condition DCs modified to promote the differentiation of Tr1 cells. Thus, similar to DCs present in gut-associated lymphoid tissue [31], [32], we show here that DCs present in the nasal-associated lymphoid tissue also play a physiologic role in the generation of regulatory T cells.

We used the *Ahr^d^*/tiger mouse to investigate the molecular mechanisms that mediate the induction of Tr1 cells by nasal anti-CD3. We found that Tr1 cells induced by nasal anti-CD3 express high levels of *ahr*, *c-Maf*, *il21* and *il21r*. Furthermore, we demonstrated that AHR and IL-21 are needed for the differentiation of Tr1 cells induced with nasal anti-CD3: Tr1 differentiation is defective in *Ahr^d^*/tiger and IL-21R deficient mice following nasal anti-CD3, and the administration of nasal anti-CD3 together with recombinant IL-21 significantly boosts the induction of Tr1 cells. These findings are consistent with our recent in vitro studies [17], [18], and demonstrate that AHR and Il-21 mediate the induction of Tr1 cells triggered by nasally administered anti-CD3. Moreover, they suggest that recombinant IL-21 can be used as an adjuvant to potentiate the induction of suppressive Tr1 cells by nasally administered anti-CD3.

**Figure 6:**
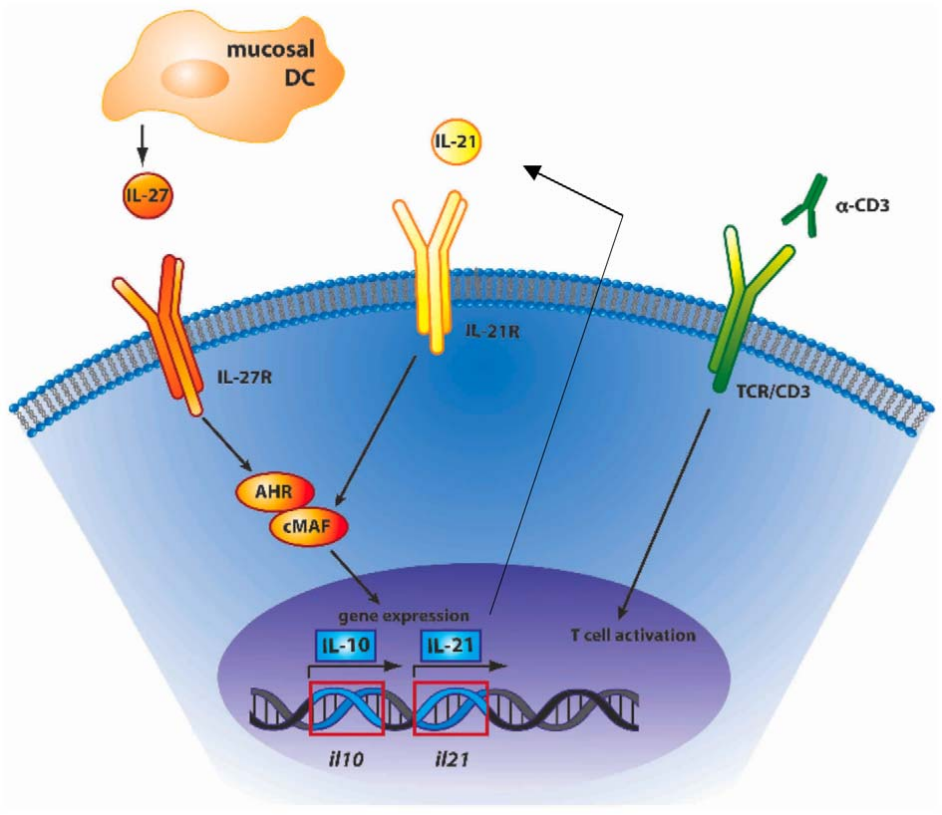
A model for Tr1 cell generation by nasal anti-CD3. Nasal anti-CD3 triggers T cell activation via TCR/CD3 complex. T cell activation in the presence of IL-27 secreted by local DCs in the CLN leads to activation of AHR and cMAF, which cooperate to transactivate the *il10* and *il21* promoters and promote IL-10 and IL-21 production. IL-21 then acts as a Tr1 growth factor in an autocrine fashion.

In summary, our data supports a model in which upon stimulation by nasal anti-CD3, resident DCs conditioned by the nasal epithelia secrete IL-27, which promotes the differentiation of Tr1 cells. IL-27R ligation triggers the synthesis of AHR and c-MAF, which then bind and transactivate the *il10* and *il21* promoters [17], [18]. Finally, IL-21 acts in an autocrine fashion to further upregulate cMAF expression thus expands Tr1 cells in vivo [13], [17], [22] (and fig.6). Our results identify a previously unknown function of mucosal DCs in the upper airways and demonstrate that nasal anti-CD3 is a unique approach to generate functional suppressive Tr1 cells in vivo that control ongoing autoimmunity. These findings identify nasal anti-CD3 as a novel therapeutic approach for the treatment of autoimmune diseases.

## Materials and Methods

### Mice

B6, MRL/lpr, DTR-GFP CD11c, Foxp3-GFP knock-in, *Ahr^d^* and tiger mice were purchased from Jackson Laboratory (Bar Harbor, Maine, USA). IL-27 receptor deficient (IL-27R^−/−^) mice were a generous gift from Vijay K. Kuchroo (Harvard Medical School). IL-21 receptor deficient (IL-21R−/−) mice were a generous gift from Warren Leonard (NIH) and Derry Roopenian (Jackson Laboratory). IL-27R^−/−^/lpr and *Ahr^d^*/tiger mice were bred and maintained at our facility at the Harvard Institutes of Medicine. Only female mice were used in lupus studies. All mice were housed in specific pathogen-free environment according to the animal protocol guidelines of the Committee on Animals of Harvard Medical School (Protocol No. 02683), which also approved the experiments.

### Antibodies, antigens and nasal treatment

Antibodies specific to CD3 (145-2c11) and CD28 (37.51) (BD Biosciences, CA, USA) were used to stimulate T cells *in vitro*. Neutralizing anti-mouse IL-10 (JES5-2A5), TGF-β (1D11), IL-4 (11B11), IL-12 (C17.8), IFN-γ (R46A2) or relevant isotype control antibodies were purchased from BioXCell, NH, USA. Neutralizing anti-mouse IL-27p28 and IL-21 antibody and mouse recombinant IL-4, IL-6, IL-12, IL-21 and TGF-β was purchased from R&D Systems, MN, USA. Fluorescent anti-mouse antibodies used in flow cytometry were CD4-specific (H129.19), CD25-specific (PC61) and IL-10 (JES5-16E3) (BD Biosciences). For Fcγ receptor blocking we used CD16/CD32-specific antibody (all from BD Biosciences). Anti-mouse LAP monoclonal antibody (16B4) was a kind gift from Taka Oida (Center for Neurologic Disease). Anti-mouse Foxp3 (FJK-16s) antibody was purchased from Ebiosciences, CA, USA. In studies of Tr1 cell generation in vivo, mice were nasally treated with 5 consecutive doses of 0.5 µg hamster IgG CD3-specific antibody (clone 145-2C11) or hamster IgG control antibody (BioXCell) dissolved in PBS. Diphtheria toxin was purchased from Sigma-Aldrich. LPS and FLT3 ligand were purchased from R&D Systems.

### T cell proliferation

Cells were cultured in triplicates at 1.5×10^6^/ml in the presence of various amounts of antibodies or alone in 96-well round bottom microtiter plates (Corning, NY, USA) for 96 hrs at 37°c with 5% CO_2_ in a humid incubator. CD4^+^ T cells were separated from murine lymphoid organs using MACS CD4 purification kit (Miltenyi Biotec). The purity of selected cells was checked by flow cytometry. In all experiments, selection efficiency was over 90%. For cell sorting CD4^+^ T cells or whole lymphocytes were stained with fluorescent anti-mouse LAP, CD4 and CD25 monoclonal antibodies (all at 0.5 µg per million cells). CD4+ CD25- or + LAP- or + GFP- or + T cells were sorted using a FACSVantage SE (BD Biosciences). The purity of each population was >95% by flow cytometric analysis. Tr1 and Th subset in vitro differentiation was carried out in the present of plate bound anti-CD3 and anti-CD28 and relevant cytokine for 48 hrs followed by passage and 2 additional rounds of 48 hr culture. Tissue culture medium was RPMI-1640 with 4.5 g/L glucose and L-Glutamine (BioWhittaker, MD, USA) supplemented with 2% penicillin and streptomycin (BioWhittaker) and 1% fetal calf serum. Cultures were pulsed with 0.25 µci tritiated thymidine ([^3^H]d Thd; PerkinElmer, MA, USA) for the last 6 hrs. [^3^H]d Thd incorporation was measured using a liquid scintillation beta counter (Wallac, PerkinElmer).

### Immunofluorescent staining

Frozen sections of cervical lymph node or spleen (5 µM) were air dried from −80°c for 30 mins and then fixed in 100% alcohol for 1 min. Sections were washed twice with PBS and non-specific binding sites were blocked with 10% normal rat serum in PBS for 1 hr at RT. Following 2 further washes with PBS, tissue sections were stained with Toprol-3 (Invitrogen, CA, USA). Tissues sections were analyzed by confocal microscopy.

### Cytokine detection

The level of cytokines produced *in vitro* by cell cultures was determined using BD OptEIA ELISA and reagent set (BD Biosciences). Samples were tested in triplicate using the manufacturer's recommended assay procedure. Cell culture supernatant was harvested at different time points (48 hrs for IL-10; 96 hrs for IL-2, IL-5, IL-12 and IFN-γ) for the detection of cytokines.

### Flow cytometry

Cells were washed (1200 RPM, 5 mins at 4°c) with PBS containing 2% bovine serum albumin in PBS (PBS/BSA, BioWhittaker). Fcγ receptors were blocked by incubation with anti-CD16/CD32 antibody for 30 mins at 4°c. Cells were washed twice before being stained with fluorescent anti-mouse cell surface molecule antibodies (1 µg/10^6^ cells/test) or relevant IC antibody for 30 min at 4°c in dark. After staining, cells were washed again with PBS/BSA before flow cytometry (FACScan^TM^, Becton Dickson, NJ, USA). For intracellular staining, cells (10×10^6^ cells/ml) in culture medium containing 1 µl GolgiSTOP (BD Biosciences) were stimulated with PMA (50 ng/ml) and ionomycin (1000 ng/ml) for 4 hrs at 37°c with 5% CO_2_ in a humid incubator. After incubation cells were fixed and permeabilized before being stained. All FACs data was analyzed using FlowJo software (TreeStar).

### Serum ELISA

Autoantibodies were measured as described previously *(14)*. Briefly, double stranded DNA (dsDNA) was used at 20 µg/ml. For the detection of total IgG, 50 µl/well of HRP-conjugated rat anti-mouse antibody (BD Biosciences) at 0.001 µg/ml was added and incubated at 37°c for 1 hr.

### Quantitative RTPCR

RNA was extracted from FACS-sorted cells or *in vitro* differentiated cells using RNAeasy columns (Qiagen, CA, USA). cDNA was transcribed as recommended (Applied Biosystems, CA, USA). The amount of cDNA was measured and equal amount of cDNA from samples was used for quantitative RTPCR. All primer/probe mixtures were obtained from Applied Biosystems. Taqman analysis was performed on AB 7500 Fast System (Applied Biosystems). Gene expression was normalized to β-actin expression.

### Adoptive transfer

To test the in vivo regulatory function of LAP+ T cells we transferred freshly isolated whole CD4^+^ or CD4^+^ T cells depleted of LAP+ cells from nasal anti-CD3 treated MRL/lpr donors to 4-wk old IL-27^−/−^/lpr recipients. Each recipient received 1×10^6^ T cells intravenously. We followed the recipients for 40 weeks and recorded their survival rate.

### Statistical analysis

Statistical differences in cell proliferation and cytokine levels were derived from 2-way ANNOVA test. We used Students'*t*-test on circulating IgG levels. The Wilcoxon rank sum test was used for all pair-wise group comparisons. A P value less than 0.05 is considered significant.
